# Integrating a tailored e-health self-management application for chronic obstructive pulmonary disease patients into primary care: a pilot study

**DOI:** 10.1186/1471-2296-15-4

**Published:** 2014-01-08

**Authors:** Viola Voncken-Brewster, Huibert Tange, Albine Moser, Zsolt Nagykaldi, Hein de Vries, Trudy van der Weijden

**Affiliations:** 1 CAPHRI, Department of Family Medicine, Maastricht University Medical Center, Maastricht, Netherlands; 2 Centre of Research on Autonomy and Participation of Persons with a Chronic Illness, Faculty of Health and Care, Zuyd University of Applied Sciences, Heerlen, Netherlands; 3 Department of Family and Preventive Medicine, University of Oklahoma Health Sciences Center, Oklahoma City, United States of America; 4 CAPHRI, Department of Health Promotion, Maastricht University Medical Center, Maastricht, Netherlands

**Keywords:** Chronic obstructive pulmonary disease, e-Health intervention, Tailoring, Self-management, Smoking cessation, Physical activity, Medication adherence, Primary care

## Abstract

**Background:**

Changes in reimbursement have been compelling for Dutch primary care practices to apply a disease management approach for patients with chronic obstructive pulmonary disease (COPD). This approach includes individual patient consultations with a practice nurse, who coaches patients in COPD management. The aim of this study was to gauge the feasibility of adding a web-based patient self-management support application, by assessing patients’ self-management, patients’ health status, the impact on the organization of care, and the level of application use and appreciation.

**Methods:**

The study employed a mixed methods design. Six practice nurses recruited COPD patients during a consultation. The e-Health application included a questionnaire that captured information on demographics, self-management related behaviors (smoking cessation, physical activity and medication adherence) and their determinants, and nurse recommendations. The application provided tailored feedback messages to patients and provided the nurse with reports. Data were collected through questionnaires and medical record abstractions at baseline and one year later. Semi-structured interviews with patients and nurses were conducted. Descriptive statistics were calculated for quantitative data and content analysis was used to analyze the qualitative data.

**Results:**

Eleven patients, recruited by three nurses, used the application 1 to 7 times (median 4). Most patients thought that the application supported self-management, but their interest diminished after multiple uses. Impact on patients’ health could not be determined due to the small sample size. Nurses reported benefits for the organization of care and made suggestions to optimize the use of the reports.

**Conclusion:**

Results suggest that it is possible to integrate a web-based COPD self-management application into the current primary care disease management process. The pilot study also revealed opportunities to improve the application and reports, in order to increase technology use and appreciation.

## Background

COPD is one of the main causes of morbidity and mortality in developed countries, causing significant economic and social burden [1]. Since COPD is not fully reversible, it is recommended that patient self-management and behavior change become an integral part of treatment, in order to prevent the disease from progressing and improve health status [2,3].

Self-management may be improved by the use of e-Health technology, an emerging tool in the field of health promotion, focusing on prevention and disease management [4], including COPD [5-7]. E-Health applications can be made accessible to many individuals at low cost, and the content can be individualized [4]. Tailoring information to patients’ needs can be attained with computer technology [8]. Computer tailoring has shown to be a promising technology to successfully promote behavior change [9,10], for instance, addressing smoking cessation, physical activity and improvement of multiple behaviors [11-15]. A computer-tailored application can be offered over the Internet to provide the patient with information to support self-management at home. Patients can access the application at any time and receive feedback on their health-related behavior and its determinants. Feedback messages are personalized and matched to patients’ needs by tailoring them to their responses [8,16].

E-Health initiatives are popular among policy makers, and many applications are being developed and implemented, at significant cost [17]. However, it is often not clear if these applications are beneficial to patients [18]. Many e-Health initiatives struggle with technology adoption [19]. One of the critical elements of optimizing the impact and uptake of an application is the involvement of stakeholders, including patients and clinicians, in the development process [19,20].

In the MasterYourBreath project (AdemDeBaas in Dutch), we developed a web-based e-Health application with computer-tailored technology that we aim to integrate into an existing primary care disease management approach. This approach was implemented in 2008 in the region of Maastricht, located in the south of the Netherlands, as an innovative disease management approach for COPD to improve quality of care and reduce healthcare cost of this growing patient population [21]. As part of this approach, COPD patients are invited for a disease management consultation with their primary care practice nurse at least once a year. Practice nurses monitor patients’ health, perform regular checks (e.g. spirometry) and aim to enhance self-management in order to change behavior, such as smoking, physical activity and medication intake.

After conducting a usability study for the MasterYourBreath application [22] we now report the results of a pilot study to assess the feasibility of integrating this application into the existing disease-management approach, by examining (1) the impact on quality of patients’ self-management and health; (2) the impact on the organization of care; and (3) the level of application use and appreciation.

## Methods

### Design

This mixed methods study applied a triangulation design model [23], in which both quantitative and qualitative data were gathered. The two types of data were integrated during the interpretation phase.

### Recruitment

Six practice nurses of primary care practices in the South of the Netherlands agreed to recruit patients who were enrolled in the COPD disease management program. Recruitment took place during patient consultations with the practice nurse. The inclusion period was planned for three months, but was extended to five months. To be included, patients had to be able to read in Dutch and have access to a computer with internet. The practice nurses received recruitment packets, which consisted of a study invitation letter, an informed consent form and a password to log in to the application. The nurses were instructed to give a short explanation of the study and hand out the recruitment packets. They then helped the patients log into the application. The nurses pre-enrolled interested patients. These patients then received a phone call from the researcher who explained the study, gave user instructions, and answered questions. If patients decided to participate, they were asked to complete a written informed consent form and send it back to the research team. The study was approved by the Medical Ethical Committee of Maastricht University Medical Centre (MUMC+).

### Intervention

#### Framework

Patients were instructed to use the application for one year ad libitum until the next yearly consultation with the nurse. We based the design of the application on applications developed in earlier studies targeting smoking cessation and physical activity [14,24]. We adapted the application for COPD patients and added a newly developed module for medication adherence. The application consists of a health risk appraisal and three behavior change modules, including smoking cessation, physical activity and medication adherence.

The application leveraged the I-Change model [25] as a theoretical framework. This model combines several theories, including the Attitude-Social influence-Self-efficacy model (ASE) [26], the Social Cognitive Theory [27], the Transtheoretical Model [28], the Health Belief Model [29], and Implementation and Goal setting theories [27,30,31].

#### Health risk appraisal

The first part of the application consisted of a health risk appraisal questionnaire to assess smoking behavior, physical activity, and medication adherence and provided the patient with feedback on their health behavior. Patient demographics were measured only at first time use. Feedback messages were based on patient input considering demographics and their behavior (smoking status, level of physical activity and level of medication adherence). Feedback also incorporated recommendations entered by the practice nurse concerning behavior change, if these recommendations deviated from the recommendations derived from the health risk appraisal questionnaire. Messages were selected through routing procedures and tailoring rules, and they were displayed on the patients’ computer screen. Patients were asked to complete the health risk appraisal every three months.

#### Behavior change modules

After receiving the health risk appraisal feedback, patients were asked to choose one behavior change objective (smoking cessation, medication adherence or physical activity) to work on over the next three months. Non-smokers and patients with a perfect score on medication adherence were not given the option to choose these modules, except if the practice nurse recommended them to the patient. Patients were able to switch modules every three months, after receiving the health risk appraisal.

Each of the behavior change modules consisted of five components: motivational beliefs, social influence, action planning, self-efficacy and maintenance. Feedback messages were tailored to patient characteristics, such as gender and age, and to key behavioral determinants based on psychosocial constructs derived from the I-Change model. Examples of these determinants are: pros and cons of physical activity, barriers to take medication according to the prescription and perceived social support to quit smoking. The most important determinants of these constructs were assessed through questionnaires that have been tested experimentally among Dutch adults in previous studies of physical activity and smoking cessation [11,32,33]. Feedback messages were further personalized by using patient names. See the ‘Feedback message’ subsection for an example of a feedback message for a patient who indicated that she did not know if she could be physically active for 30 minutes a day during the winter and has not made any plans that could help her to be physically active during the winter time.

A more detailed description of the modules can be found elsewhere [22].

#### Feedback message

You indicated that you don’t know if you will be able to be physically active for 30 minutes a day during the winter months. People do fewer activities outside during the winter, it is often very cold, so it is understandable if you don’t manage to get enough physical activity every day. However, the period of inactivity will be very long, if you are not active all winter.

You said that you don’t have any plans that help you get enough physical activity during the winter, so it might be a good idea to think about it. Here are some examples of plans you could make:

•Dress warmly before you go outside. Start slowly and warm up your muscles before you go.

•Sign up at a gym, so you can be physically active and exercise inside.

•Plan to be physically active at home. Certain exercises, such as flexibility and muscle strengthening exercises can be done at home.

•If you exercise outside, breathe in through your nose and a scarf, so the cold air will be warmed up before it enters your lungs.

#### Nurse feedback

Practice nurses received a report once every three months. The report included information on how many times a module was used, motivational beliefs (perceived pros and cons of the behavior change), perceived social influence, action plans made to prepare behavior change and self efficacy (perceived barriers).

### Data collection

Data collection started in April 2010 and ended in September 2011. Data were collected at baseline, after the patient’s consultation with the practice nurse, and approximately one year later, through the self-reported health risk appraisal questionnaire at the first time and last time use of the application. Data from medical records entered by the nurse at the consultations pre- and post-intervention were abstracted. Semi-structured face-to-face interviews were conducted by two researchers (V. Voncken-Brewster (VV) and A. Moser (AM)) during the second half of the intervention period with patients and the practice nurses. Interviews with patients (Additional file 1) took between 25 and 45 minutes and interviews with nurses (Additional file 2) approximately 20 minutes. All nurses were asked to keep a record of reasons for patient non-participation. In addition, VV conducted field visits to the practices and called the practice nurses to discuss and solve recruitment issues.

### Reminders

Patients received an average of 12 prompts to log into the application over the intervention period and they received two reminders to fill out the post-intervention questionnaire. Most patients received the reminders through email, except for one patient who did not use e-mail and so received reminders through regular mail.

### Measures

Demographic variables that were measured in the questionnaire were: age, gender, marital status, nationality, education level and current work status. Body mass index (BMI), the severity of the disease according to the standards defined by the Global Initiative for Chronic Obstructive Lung Disease (GOLD) [34] and FEV1 (% predicted) post bronchodilator (PB) were retrieved from medical records.

#### Patient self-management and health status

To uncover the level of patients’ self-management and health status both quantitative and qualitative measures were used. *Smoking* was measured using the questionnaire by asking patients if they smoked, what they smoked (cigarettes, rolling tobacco, cigars, or pipe tobacco), and how much they smoked. We relied on self-report since Wilson et al. [35] show that the majority of COPD patients report their tobacco consumption reliably. *Physical activity* was measured in minutes of moderate and intense physical activity a week by the Short Questionnaire to Assess Health-Enhancing Physical Activity (SQUASH) [36]. *Medication adherence* was assessed by the abbreviated version of the Medication Adherence Rating Scale (MARS-5) (scoring 5 – 25, higher score means better adherence) [37]. Besides patient self-report, practice nurses’ assessment of patients’ smoking behavior (number of cigarettes a day), physical activity (number of days a week), and medication adherence (poor, score = 1/moderate, score = 2/good, score = 3) were obtained from patient medical records.

Patients’ health status was measured by the Medical Research Council (MRC) Dyspnea Score (scoring 1 – 5, higher score means worse dyspnea) [38] and FEV1 (% predicted) post bronchodilator (PB). These data were obtained from medical records. Disease-specific quality of life was assessed in the patient self-reported questionnaire using the Clinical COPD Questionnaire (CCQ) (scoring 0 – 6, higher score means worse quality of life) [39].

Qualitative data about the influence of the application on smoking, physical activity and medication adherence were collected through interviews with patients.

#### Organization of care

Qualitative information was collected regarding the impact on the organization of care. Practice nurses were asked about if and how the integration of the application impacted the organization of care and about their use and appreciation of the reports.

#### Application use and appreciation

Quantitative data were collected about application use, including frequency of use during the intervention period, frequency of use of each behavior module and completion time.

Qualitative information was gathered during interviews with patients, through questions about their use and satisfaction with the application.

### Analysis

#### Quantitative analysis

Descriptive statistics, including number (%) for categorical variables and median (range) for numerical variables, were calculated for demographic characteristics at baseline. The median (range) of self-management and health status-related variables were calculated for complete cases pre- and post-intervention. Also, the median (range) of the within-person difference between pre- and post-intervention were calculated for these variables. All incomplete cases were excluded. Application use, completion time and frequency of use of each behavior module were calculated for all cases. Sessions were excluded if patients logged on, but discontinued the session before receiving feedback.

#### Qualitative analysis

Field notes of visits with nurses to discuss recruitment issues and nurses’ records of documented reasons for patients’ non-participation were reviewed. Interviews were audio taped and transcribed verbatim. Content analysis was performed using the constant comparative method [40]. The interviews were coded by VV. The transcripts were read and re-read. Descriptive codes were assigned. The descriptive codes were compared and contrasted to simultaneously define and refine properties, subcategories and categories. In analytical sessions the two researchers who performed the interviews discussed the coding and analysis frequently to aid data interpretation and formulate the findings.

## Results

### Participants

Of the six nurses participating in this study, only three recruited patients for the study. One nurse did not have any disease management consultations during the study period. The other five practice nurses had a total of 118 COPD patient consultations. Sixteen patients (13.6%) were recruited and 11 patients (9.3%) participated. Although nurses said that the recruitment procedure was easy and not time consuming, they did not consistently remember to ask patients or decided not to ask certain patients if they thought that they would not want to participate. Reasons mentioned by patients for not participating included having no time, not needing lifestyle advice, not having access to a computer with internet and personal reasons.

Three patients dropped out of the study during the intervention period (the first had heart surgery, the second did not want to continue due to advanced age, and the third was referred to a lung specialist). The medical records of all 11 patients were therefore available for analysis pre-intervention, and the records of eight patients were available post-intervention. Seven patients participated in an interview; the eighth patient declined. Four patients completed the post-intervention questionnaire. The nurses who pre-enrolled patients participated in an interview. See Table 1 for the characteristics of all patients at baseline.

**Table 1:** Baseline characteristics of patients

**Variable**	**N = 11**
**Age** (range), yr	Median = 56 (39 to 82)
**Gender**	
Male	8 (72.7%)
Female	3 (27.3%)
**GOLD**	
Stage 0	1 (9.1%)
Stage 1	3 (27.3%)
Stage 2	7 (63.6%)
**FEV1% post-BD** (range)	Median = 64.3 (45.2 to 74.0)
**BMI** (range)	Median = 26.2 (17.3 to 31.4)
**Marital Status**	
In a relationship	1 (9.1%)
Married	9 (81.8%)
Divorced	1 (9.1)
**Nationality**	
Dutch	11 (100%)
**Education level**	
Primary school/basic vocational school	3 (27.3%)
Secondary vocational school/high school degree	7 (63.6%)
Higher professional degree/university degree	1 (9.1%)
**Current work status**	
Employed	8 (72.7%)
Not employed	3 (27.3%)

### The impact on patients’ self-management and health status

#### Quantitative results

##### Smoking

Five patients were smokers at the beginning of the study. Pre and post-intervention data on the number of cigarettes were available through medical records of four patients. None of the four patients quit smoking, but the number of cigarettes smoked a day decreased slightly (Table 2).

**Table 2:** Results of patients’ self-management and health status of complete cases

**Measure**	**Pre-intervention median (range)**	**Post-intervention median (range)**	**Within-person difference median (range)**
***Smoking***			
cigarettes/day (n = 4)	20.0 (10.0 to 40.0)	15.0 (10.0 to 40.0)	−2.5 (0.0 to 5.0)
***Physical activity***			
days/week (n = 8)	5.0 (0.0 to 7.0)	5.0 (0.0 to 7.0)	0.0 (−5.0 to 5.0)
SQUASH (minutes/day) (n = 4)	336.0 (54.0 to 564.0)	347.5 (132.0 to 540.0)	−39.0 (−66.0 to 161.0)
***Medication adherence***			
Poor/moderate/good (scoring 1 to 3) (n = 8)	3.0 (2.0 to 3.0)	3.0 (2.0 to 3.0)	0.0 (−1.0 to 1.0)
MARS5 (scoring: 0 to 25) (n = 4)	23.5 (23.0 to 24.0)	23.0 (20.0 to 25.0)	−0.5 (−4.0 to 2.0)
***Health status***			
FEV1% post-PB (n = 8)	66.2 (45.2 to 73.6)	63.1 (46.3 to 72.4)	0,4 (−7.4 to 4.3)
MRC (scoring: 1 to 5) (n = 5)	1.0 (1.0 to 2.0)	1.0 (1.0 to 1.0)	0.0 (−1.0 to 0.0)
CCQ (scoring: 0 to 6) (n = 4)	1.0 (0.5 to 1.6)	0.8 (0.1 to 2.4)	−0.2 (−0.4 to 0.8)

##### Physical activity

Medical records (n = 8) showed that patients increased the number of days per week that they were physically active. However, the median within-person difference score on the SQUASH (n = 4) showed a decrease in minutes of physical activity per day (Table 2).

##### Medication adherence

No difference was found in adherence to medication prescriptions, reported as poor/moderate/good, according to medical records. The score on the MARS-5 (n = 4) decreased slightly (Table 2).

##### Health status

CCQ scores decreased slightly, indicating an improvement in quality of life. No difference was found in MRC dyspnea scores. A small increase in the FEV1% post-PB was indicated by the within-person difference median (Table 2).

#### Qualitative results

Four of the seven interviewees said that the application influenced their smoking behavior, physical activity or medication adherence.

‘The application helped, because I am smoking less’

‘*I have this physical activity class with the physical therapist now once a week’*

Two patients, however, thought that the feedback was not applicable to them since they already maintained a healthy lifestyle. One patient did not use any of the behavior modules at all. He did not expect them to be useful, since the baseline health risk appraisal questionnaire and feedback did not provide useful information to him.

‘I think this is more for people who are less physically active. The feedback is good for these people and for people who smoke of course’

‘I expected that the study could help me quit smoking, but I could not get that out of this.’

Patients explained how the application helped changing their behavior. One patient said that it provided support to prevent smoking relapse*.*

‘You can find some support if you relapse in smoking and maybe the application will guide you’

The application made patients more aware of their behavior.

*‘Just being active at work is not enough. That has to be more. Actually, I learned this from the application’*.

Two patients mentioned that is was useful to be reminded to change their behavior, while two other patients disputed the need for reminders to quit smoking, because they were aware of that*.*

‘Every time I will be reminded that you should take your medication on time, it says it in the feedback, I like that’

One patient mentioned that the application helped her cope with difficult situations, in which it is hard not to smoke.

### The perceived impact on the organization of care

#### Qualitative results

A perceived benefit on the organization of care as reported by a patient was that an additional practice visit might have been replaced by the use of the application.

‘The nurse told me: you can come to the practice, to see her, or you can do this on the computer’

Practice nurses said that a computer application could be beneficial to patients, but they thought that it would only be suitable for a subset of their patients, as other patients may prefer face-to-face contact.

‘A practice nurse should be able to provide interventions. There is a diversity of people, some people want to talk face-to-face and some really want to do it themselves, in that case you can offer the application as support’.

Another perceived benefit was that the application elicited an active self-management role for the patient. Considering the reports, nurses liked that they were informed about the patients’ progress. They did not invite patients back earlier for a consultation than planned based on the reports, but discussed it at the next consultation.

‘I think it is interesting to receive feedback about patients and I talk to them about it during the next consultation.’

The content of the reports was informative and not too long.

‘It is nice to see the information compact.’

All components of the reports were considered useful, except for the part about social influence, as they thought that this would only be important for the patient to know and not for the nurse. Reports were not specifically discussed during the consultations, but nurses kept them in mind, while talking to the patient. They were very similar to what was normally discussed during a consultation.

‘I respond to what patients are saying with the reports in the back of my mind.’

Nurses suggested integrating the reports directly into the patients’ electronic medical record, for a better work-flow.

‘It would be nice if it would be integrated in the electronic medical record, so you can, with one click, without going to an email account, see it right away. That’s were it belongs mostly in my opinion.’

### Application use and appreciation

#### Quantitative results

**Figure 1:**
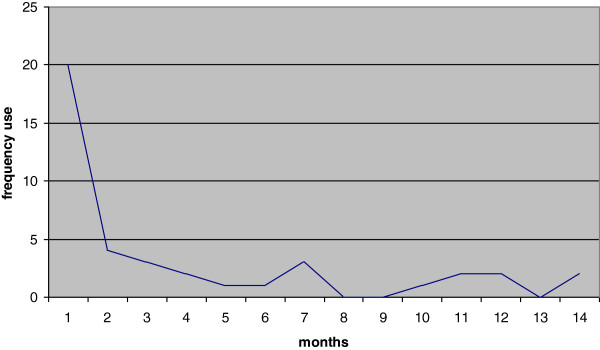
Frequency of application use of 11 patients during the intervention period.

Patients used the self-management application a median of 4 (1 to 7) times. Use was highest during the first month, indicating a total of 20 times used by 11 patients; early use included completing the Health Risk Appraisal questionnaire and receiving feedback, as well as the behavior change modules. Application use decreased to 0–4 times a month in the following months (Figure 1). In total, the medication adherence module was used 12 times, the smoking cessation module 2 times and the physical activity module 9 times. The median time for patients to complete a session, was 16 minutes (3 minutes to 95 minutes).

#### Qualitative results

Three out of six patients, who used at least one of the behavior change modules, found that the information was adequately personalized and all of them would recommend the application to others.

‘Interviewer: Would you recommend it to others? Patient: Yes, because it is useful. If you fill it out, then it points out things that are important.’

Patients liked that they could print the feedback. Patients also mentioned aspects that they did not appreciate. Three patients thought the questionnaires were clear, while others perceived them as difficult or preferred open-ended questions. One patient said that the questionnaires were too long. Four patients who used at least one of the modules said that the information given by the application was not new to them. It bothered one patient that the application would consider behavior at the moment of use and not one’s usual behavior. For example, factors such as bad weather and occasional illnesses were not taken into account*.*

‘The thing I didn’t like about the application was that when I fell off the stairs and had my ribs bruised, I wanted to go for a walk, but I couldn’t. So using the application at such a moment… You should fill it out when you are going to do physical activity again … I think that everybody has that in the winter, that you are less physically active.’

Four patients thought the feedback concerning medication adherence was too strict. One patient emphasized the irrelevance of the social influence component.

‘What has my family to do with it? It is my life.’

Reasons mentioned for discontinuing use of the application were lack of variation, forgetting the web-address or password and time constraints.

‘You won’t get new information and it takes time’

Providing reminders by sending out prompts and a pen and notepad with the project logo motivated patients to log into the application. A practice nurse said that patients chose the smoking cessation module the least, presuming that smoking is the most difficult behavior to change.

‘The hardest module, smoking cessation was chosen relatively little. Well, I just assume that this is the hardest.’

## Discussion

In this study we assessed the feasibility of integrating a computer-tailored self-management application for COPD patients into Dutch primary care practices. The results of this pilot study suggest that integrating the application may be beneficial to patients and the organization of care. Several patients reported that it helped them quit smoking, increase their level of physical activity and adhere to their medication regimen. Interviews with practice nurses and patients revealed a beneficial impact on the organization of care, such as being able to monitor patients’ progress in self-management between consultations and stimulating patients to assume an active role in self-management, with limited effort on the part of the practice nurse. The intervention did not aim to replace practice visits in order to improve efficiency of care. Instead, our intervention intended to improve the quality of care by providing patients with a self-management tool at home when they did not have the immediate support of the nurse. Yet, an additional practice visit with a patient might have been replaced by the use of the computer application.

The results of the pilot study also revealed several challenges, the main one being the low number of revisits to the application explained, in part, by patients’ desire for more variation in the application to sustain their interest. This is a common problem in e-health research [41]. Refreshing content on a regular basis could promote revisits. Another way is sending more effective prompts with an optimal prompt frequency [42-44]. A study of Schneider et al. [44] found that sending prompts two weeks after the initial visit is more effective than a longer time period. However, this study focused only on the first revisit and it is not clear if having a short time interval and consequently a higher prompt frequency over a longer period of time would be as effective. Further research is needed considering revisits over a longer time period to determine the optimal prompt frequency. Another way to improve application use could include the option to choose elective components instead of an entire module, in order to adjust the content and the time spent per visit to patient preference. This could add to the perception of variation and result in a higher level of appreciation. In general, giving the patient more control over the time he or she spends using the application could encourage utilization, since length and information overload seem to be important reasons for an individual to quit using an online application [45,46]. Another strategy to promote revisits could include the opportunity for patients to monitor their goal achievement over time and send prompts to revisit and evaluate these goals at the appropriate time when the patient anticipated achieving the goal.

Patients also addressed some aspects of the application that they did not appreciate, which may lead to less utilization. For example, one patient did not appreciate the social influence component. Since social influence is thought to be an important predictor of behavior [27,47], we decided not to remove this component. However, letting the patient choose elective components and not forcing the patient to use the entire module can make the application more appealing, as patients can skip unappealing components based on their preference. Practice nurses indicated that it would improve their work-flow if the reports were included in the electronic medical record. Receiving the reports through the electronic medical record may prompt the nurses to discuss the report with the patient during the consultation, which might improve patient satisfaction with the consultation [48].

We encountered considerable recruitment problems in the course of the study. We chose a visit-based recruitment method since it was congruent with the nature of a practice-based study. Furthermore, it was thought to have a number of benefits, including the nurse-patient relationship, which could increase the enrollment of individuals with lower levels of education and those with low motivation to change their lifestyle. These individuals are often underrepresented in computer-tailoring studies [49]. However, this was not the case in our study. Nurses forgot or were reluctant to invite patients they expected would not participate. Prompting nurses through the electronic medical record to remind them to enroll patients for the study at the time of the consultation might increase the number of participants and might also increase enrollment of usually underrepresented patient groups [50].

Patient recruitment issues resulted in several limitations. Some of our results might be biased, because it is likely that only more motivated nurses and patients participated. Other differences in characteristics between participants and patients who did not participate, such as educational status and disease severity, could also have contributed to biased results. However, we were not able to gauge the differences between these groups, since we could not collect data on patients who did not participate. In addition, the small sample size did not allow us to evaluate quantitative outcomes to measure the effect of the intervention on health and health-related behavior of the patients that we may use for sample size calculation in a Randomized Controlled Trial. Most of our conclusions on the feasibility of the application and its integration in primary care are based on qualitative data. However, the combination of quantitative and qualitative measures provided complementary information and valuable insight into the strengths and limitations of our approach. Lessons learned may also help us improve the application and its integration into primary care.

## Conclusion

In conclusion, while the use of this patient self-management tool could potentially enhance self-management and the organization of care, our e-Health application requires several strategic improvements to make it more appealing to both patients and practice nurses.

## Competing interests

Hein de Vries is scientific director of Vision2Health, a company that licenses evidence-based innovative computer-tailored health communication tools.

## Authors’ contributions

HT, TvdW, HdV and VV designed the study. VV conducted the study and developed the application. HdV, TvdW, AM and HT contributed to the content development and screened the application and AM contributed to the qualitative data collection. VV analyzed the data and AM and ZN were significantly involved in the analysis and interpretation phase. VV drafted the manuscript. All authors were involved in revising the manuscript critically and approved the last version.

## Pre-publication history

The pre-publication history for this paper can be accessed here:

http://www.biomedcentral.com/1471-2296/15/4/prepub

## Supplementary Material

Additional file 1 Semi-structured interview questionnaire with patients. Click here for file

Additional file 2 Semi-structured interview questionnaire with nurses. Click here for file
