# Supplementary material for: Integrating a tailored e-health self-management application for chronic obstructive pulmonary disease patients into primary care: a pilot study
Source: BMC Fam Pract. 2014 Jan 8;15:4. doi: 10.1186/1471-2296-15-4 (PMC3907149; doi:10.1186/1471-2296-15-4)
Supplement: Additional file 1 — Semi-structured interview questionnaire with patients. [file 1471-2296-15-4-S1.doc]

### Additional file 1.

**Semi-structured interview questionnaire with patients**

1. Did you appreciate receiving a phone call prior to starting MasterYourBreath at home? *Ask more about why.*

2. How long after the phone call did you start using MasterYourBreath?

3. When you first used MasterYourBreath, could you use the first questionnaire about health and lifestyle without any problems? *If not:* What happened?

4. What did you think about this questionnaire? *If not mentioned ask more about clarity, length and level of difficulty.*

5. What did you think about the short feedback message that followed the questionnaire? *If not mentioned ask more about whether the advice was encouraging or not, correct or incorrect, useful or not and peasant or not.*

6. What did you think of the traffic lights (as part of the advice)?

7. Did you start one of the behaviour modules for smoking, physical activity or medication adherence?

8. Were you satisfied with the behaviour you chose, or did you wish you had chosen a different behaviour? *If not mentioned: Why or why not?*

9. Did you choose a different behaviour later on?

10. Did you think the feedback messages concerning *(fill in the chosen behaviour)* were useful? *If not mentioned: Why or why not?*

11. Did the feedback messages contain new information for you? *Ask for examples*

12. Did the feedback messages make you want to quit smoking/be more physically active/adhere better to your medication prescriptions *(fill in the chosen behaviour*)?

13. Did you feel like the feedback messages were personally written for you?

14. How often did you use MasterYourBreath? *If not mentioned:* *Why did you use it … times?*

15. Did you receive reminders of MasterYourBreath?

16. What did you think of the content of the reminders?

17. What changes would you make to the content?

18. Did you think that you received the reminders too often? *If not mentioned: why or why not?*

19. How often would you have liked to receive a reminder?

20. Would you have preferred to be reminded in a different way? *If not mentioned: How?*

21. Did you receive the MasterYourBreath pen and notepad? *If not mentioned: What did you think of this?*

22. Did you find MasterYourBreath useful? *If not mentioned: Why or why not? Can you name an example?*

23. Would you recommend MasterYourBreath to others? *If not mentioned: Why or why not?*

24. For what group of people could MasterYourBreath be useful?

25. What would an ideal program to help you with your lifestyle look like? *Ask about movies/support group/dairy/chosing which information you want to read/new content/quiz etc.*
