# Supplementary material for: Integrating a tailored e-health self-management application for chronic obstructive pulmonary disease patients into primary care: a pilot study
Source: BMC Fam Pract. 2014 Jan 8;15:4. doi: 10.1186/1471-2296-15-4 (PMC3907149; doi:10.1186/1471-2296-15-4)
Supplement: Additional file 2 — Semi-structured interview questionnaire with nurses. [file 1471-2296-15-4-S2.doc]

**Additional file 2**

**Semi-structured interview questionnaire with nurses**

1. What was your experience with participating in the MasterYourBreath study?

2. What aspects were good?

3. What can we improve? Or what aspects were not good? *If* *not mentioned during question 2 or 3* *ask about study information, user friendliness, a tool to talk about self-management behaviour during a consultation, availability of the researcher, patient inclusion procedure*

4. How many patients of your practice participated?

5. Did you receive the feedback reports concerning your patients’ use of MasterYourBreath?

6. Can you remember how many you received?

7. Have you read all reports? *If not mentioned: Why or why not?*

8. What did you think of the feedback reports? *If not mentioned ask about content, usefulness for motivating lifestyle change, knowledge about what patients do at home about their lifestyle.*

9. What did you think about receiving reports through e-mail?

10. Did you look at the feedback reports during the consultation with the patient? *If so: How did you use the feedback report during the consultation? If not: Why not?*

11. How did the patient respond to this?

12. Can you advise us on what we can do to improve the use of feedback reports? *If not mentioned ask about delivering the reports through medical records or patients bringing the report to the consultation.*

13. What did you think of the length of the feedback reports?

14. Is anything missing in the feedback reports? *Ask if all components of the reports were sufficiently described*

15. The plan for pre-enrolling patients for the study was that patients would be invited during the consultation and you and the patient log in together and fill out the patient’s contact information. How did this go? Did you experience any problems?

16. Did you invite every COPD patient who came to the consultation?

17. If not, what was the reason for not inviting these patients?

18. How many patients do you estimate came for a consultation and how many did you invite to participate in MasterYourBreath?

19. What was your experience with asking patients to participate in MasterYourBreath? *If not mentioned ask: was it easy or hard and why?*

20. What were patients’ reasons for not participating?

21. Did the researcher make you feel involved in the study? What made you feel involved or not involved?

22. Would you have liked for the researcher to have kept you more involved in the study? *If yes: In which way?*

23. *Explain that they were not specifically instructed to do the following, but:* Did you encourage participating patients to use MasterYourBreath?

24. *If yes:* Can you tell us more about this?

25. Have you asked patients to come for a consultation earlier than planned after reading a feedback report?

26. How much time did the MasterYourBreath project take you per patient? *Remind about pre-enrollment and discussing feedback reports.*

27. Do you think that MasterYourBreath contributes to better health care? *If yes: in what way? If no: why not/what would have to change?*

28. Do you have any final thoughts or comments for further research?
